# Levels of Interferon-Gamma Increase after Treatment for Latent Tuberculosis Infection in a High-Transmission Setting

**DOI:** 10.1155/2012/757152

**Published:** 2012-12-17

**Authors:** Iukary Takenami, Brook Finkmoore, Almério Machado, Krisztina Emodi, Lee W. Riley, Sérgio Arruda

**Affiliations:** ^1^Advanced Laboratory of Public Health, Oswaldo Cruz Foundation, Gonçalo Moniz Research Center, Salvador, Bahia 40296 710, Brazil; ^2^Division of Infectious Diseases & Vaccinology, School of Public Health, University of California, Berkeley, CA 94720, USA; ^3^Bahia School of Medicine and Public Health, Salvador, Bahia 40290 000, Brazil; ^4^Hospital Especializado Octávio Mangabeira, Secretary of Health of Bahia State, Salvador, Bahia 40320 350, Brazil

## Abstract

*Objectives*. We investigated IFN-**γ** levels before and after a six month course of isoniazid among individuals with latent tuberculosis infection (LTBI) in a high-transmission setting. *Design*. A total of 26 household contacts of pulmonary tuberculosis patients who were positive for LTBI by tuberculin skin test completed six months of treatment and submitted a blood sample for a follow-up examination. The IFN-**γ** response to *Mycobacterium tuberculosis*-specific antigens was measured, and the results before and after the completion of LTBI treatment were compared. *Results*. Of the 26 study participants, 25 (96%) showed an IFN-**γ** level higher than their baseline level before treatment (*P* ≤ 0.001). Only one individual had a decreased IFN-**γ** level after treatment but remained positive for LTBI. *Conclusion*. In a high-transmission setting, the IFN-**γ** level has increased after LTBI treatment. Further studies must be undertaken to understand if this elevation is transient.

## 1. Introduction

Commercially available interferon-gamma release assays (IGRAs) diagnose *Mycobacterium tuberculosis (M. tb) *infection by measuring interferon-gamma (IFN-*γ*) released by cells of whole blood after *in vitro* stimulation with *M. tb*-specific antigens, early secreted antigenic target 6 (ESAT-6), and culture filtrate protein 10 (CFP-10) [1]. These diagnostic tests are more specific than the tuberculin skin test (TST) because they include *M. tb*-specific antigens encoded by the region of difference 1 (RD1) which is absent from *Bacillus* Calmette-Guérin (BCG) and most nontuberculous mycobacteria (NTM) [1–3]. A recent meta-analysis concluded that the commercially available IGRAs have excellent specificity to diagnose latent tuberculosis infection (LTBI) and are unaffected by BCG vaccination [4]. 

The capacity of IGRAs to monitor the treatment response tuberculosis (TB) and LTBI is under investigation. This study was motivated by studies which have found that the level of IFN-*γ* released by cells of whole blood after *in vitro* stimulation with *M. tb*-specific antigens in commercial IGRAs declines in patients treated with multidrug regimens for active TB [5–7]. Despite these results, some investigators have concluded that IGRAs may not be helpful in monitoring TB treatment because of high intersubject variability and because test reversion is rare [8, 9].

In contrast to treatment of active TB, previous studies on T-cell response before and after treatment for LTBI have shown conflicting results [10–12]. A study from Japan found that the levels of IFN-*γ* decreased after LTBI treatment although the commercial IGRA result did not revert to negative [11]. Another study in health care workers in India found that IFN-*γ* levels remain high after LTBI treatment [10]. In Singapore, a study found that LTBI treatment had a differential effect on T-cell responses depending on which RD1 antigen the T-cells were exposed to [12]. It is unclear if the amount of IFN-*γ* released by T-cells stimulated with the *M. tb*-specific antigens in commercial IGRAs follow a specific pattern after completion of LTBI treatment. Moreover, it is unknown if specific T-cell responses after LTBI treatment are different in settings where the transmission of *M. tb* in the community is low compared to setting where transmission is high [10, 13–16]. This study compares the IFN-*γ* levels, measured by a commercial IGRA, of household contacts (HHCs) of pulmonary TB patients before and after six months of isoniazid (H) for the treatment of LTBI in a high-transmission setting.

## 2. Study Population and Methods

### 2.1. Setting

 Participants were recruited from Hospital Especializado Octávio Mangabeira (HEOM) a 217-bed public chest-disease hospital in Salvador, Brazil. In 2007, Salvador had a TB incidence of approximately 79 per 100 000 population [17].

### 2.2. Study Participants

Study participants were HHCs of patients hospitalized with pulmonary TB at HEOM who tested positive for LBTI by TST and completed a six-month course of H [18]. None of the index cases or HHC were taking medication for the management of HIV. The characteristics of the index cases and all HHC are described elsewhere [19]. The characteristics of the HHC who initiated LTBI treatment and their adherence to the six-month regimen are described elsewhere [20]. It is well documented in the literature that HHC with LTBI are at high risk of developing active disease during the two years after infection [21, 22]. 

The study was approved by the human subjects committees of the Oswaldo Cruz Foundation in Salvador, Brazil, and the University of California at Berkeley, USA. Informed consent was obtained for all study participants.

### 2.3. Data Collection

Study participants underwent a baseline examination (TST, blood test, and interview) as part of another study [19]. LTBI could only be diagnosed in those who returned to have their TST read. Those eligible for LTBI treatment were offered the six-month supply of H free of charge [20]. Among the HHC who initiated LTBI treatment, HHCs considered to have completed treatment were those who collected six supplies of 30 H tablets from HEOM. A member of the study team (IT) called all HHC who completed LTBI treatment and invited them to return to HEOM for a follow-up examination and blood draw. HHCs were called and asked to participate in the follow-up examination a maximum of four times.

### 2.4. Treatment

Study participants were given six months of daily H treatment (5 mg/kg, up to 300 mg daily); this is the standard treatment regimen in Brazil [18]. 

### 2.5. Laboratory Tests

The TST was administered according to the Mantoux method, by injecting intradermally 2 tuberculin units (in 0.1 mL) of purified protein derivate (RT23 PPD; Staten Serum Institute, Copenhagen, Denmark). TST reaction was 72 hours after an administration by the chest physician on the study team (AMJ). The cut-off point for a positive reaction was ≥10 mm induration because this was the cutoff used in the decision to initiate treatment in Brazil at the time of the study, according to the Brazilian Society of Thoracic and Phthisiology [18].

The blood was examined with a commercially available IGRA, the QuantiFERON-TB Gold In Tube (QFT-IT; Cellestis Limited, Carnegie, VIC, Australia). QFT-IT includes the following *M. tb*-specific proteins: secreted antigenic protein 6 kDa (ESAT-6), culture filtrate protein 10 kDa (CFP-10), and TB7.7 (Rv 2654). The test was performed according to the manufacturers instructions at the immunology laboratory at Gonçalo Moniz Research Center in Salvador, Bahia, Brazil. The cut-off value for a positive response was 0.35 IU/mL. Samples that gave indeterminate results were reprocessed. Blood was drawn for the baseline IGRA before the TST was administered; both were conducted on the same day. 

### 2.6. Statistical Analysis

 Data were analyzed using GraphPad Prism v.5.0 (GraphPad Inc., San Diego, CA, USA). Wilcoxon signed rank test was used to compare the median IFN-*γ* levels before and after H treatment. The difference between the median values at the two time points was considered statistically significant when the *P* value ≤0.05. 

## 3. Results

**Figure 1:**
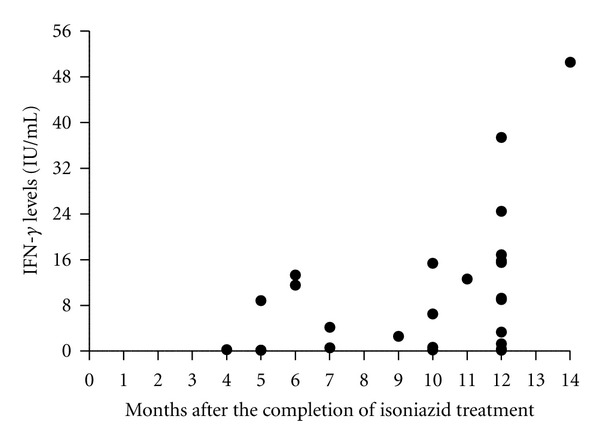
The difference between the baseline and follow-up IGRA result according to the number of months elapsed between the completion of H treatment and the follow-up examination. IFN-*γ* levels were measured by QFT-IT and plotted for each study participant (*n* = 26). H = isoniazid; IFN-*γ* = interferon-gamma; QFT-IT = QuantiFERON TB Gold in Tube.

Of the 101 HHC of pulmonary TB patients who tested positive for LTBI by TST and initiated on LTBI treatment between January 2007 and February 2008, 55 (54.5%) completed six months of therapy with H. Of the 55 HHC, 26 (47.3%) returned to HEOM for the follow-up examination and submitted a second blood sample for a second test. The second blood sample was submitted for follow-up examination between four and 14 months after the completion of LTBI treatment (Figure 1). None of the 26 HHC who returned for the follow-up examination sought medical attention at HEOM for symptoms consistent with TB between the time they concluded LTBI treatment and the follow-up examination. 

The median age of the 26 HHC who returned for the follow-up examination was 27 years (IQR 12.0–37.5). Sixteen (61.5%) were women, and 23 (88.5%) had BCG vaccination scars. At baseline examination, the median value of the TST induration was 13 mm (IQR 12.0–16.7) among the 26 HHC who returned for follow-up examination. Of the 29 HHC who did not return for follow-up examination, the median age was 19 years (IQR 11.5–39.0); 15 (51.7%) were male and 28 (96.5%) had a BCG vaccination scar, and the median value of the TST induration was 16 mm (IQR 11.0–19.5) at baseline examination. The sociodemographic, clinical, and laboratory profile of the HHC who did and did not return for follow-up examinations were not significantly different (data not shown). 

**Figure 2:**
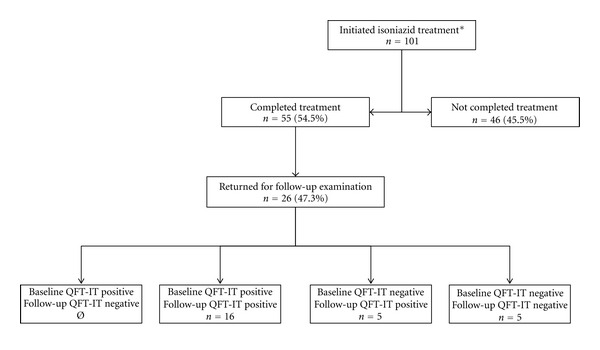
Flow chart of the study population and the study participants. *TST ≥ 10 mm. H: isoniazid; QFT-IT: QuantiFERON TB Gold in Tube; TST: tuberculin skin test.

Of the 26 HHC who completed H treatment and returned for follow-up examination, 16 (61.5%) were QFT-IT positive, and 10 (38.5%) were QFT-IT negative at baseline examination. All 16 HHC who were QFT-IT positive at baseline tested positive by QFT-IT in the follow-up examination. Of the 10 HHC who were QFT-IT negative at baseline, five (50%) tested negative and five (50%) tested positive by QFT-IT in the follow-up examination. Not one HHC who tested positive by QFT-IT at baseline tested negative after completing H treatment (Figure 2). 

**Figure 3:**
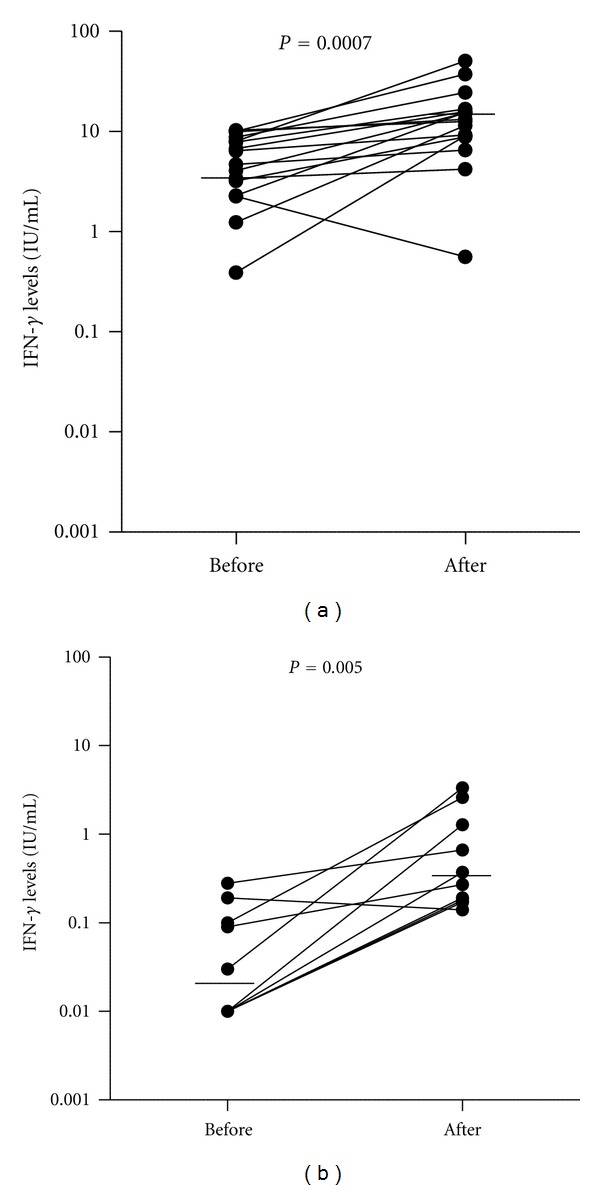
IFN-*γ* levels before and after H treatment for LTBI measured by QFT-IT stratified by baseline QFT-IT result; (a) positive QFT-IT and (b) negative QFT-IT. The solid line represents the median value of IFN-*γ* levels at the two time points. The differences between the median values in each group before and after H treatment are significant by the Wilconxon's signed rank test (*P* = 0.0007 and *P* = 0.005, resp.). IFN-*γ*: interferon-gamma; H: isoniazid; QFT-IT: QuantiFERON-TB Gold in Tube.

Among the 16 HHC who tested positive by QFT-IT before LTBI treatment, the median IFN-*γ* level to the *M. tb*-specific antigens at baseline evaluation was 5.55 IU/mL; the median IFN-*γ* level of this group after six-months of H treatment was 12.97 IU/mL (*P* value = 0.0007) (Figure 3(a)). One individual who tested QFT-IT positive before LTBI treatment had a decreased IFN-*γ* level after treatment but remained QFT-IT positive (IFN-*γ* ≥ 0.35 IU/mL) (Figure 3(a)). Among the 10 HHC who tested negative by QFT-IT before LTBI treatment, the median IFN-*γ* level to the *M. tb*-specific antigens at baseline evaluation was 0.02 IU/mL; the median IFN-*γ* level of this group after six months of H treatment was 0.32 IU/mL (*P* value = 0.008) (Figure 3(b)). 

The difference in the median value of IFN-*γ* before and after treatment was 7.42 IU/mL and 0.30 IU/mL in HHC who were QFT-IT positive and those negative at baseline, respectively (*P* value = 0.0012).

## 4. Discussion

The treatment of LTBI is a basic strategy for TB control. A six-month course of a single drug, H, is effective in preventing individuals with LTBI from progressing to active disease [23]. It is difficult, however, to monitor LTBI treatment in individual patients. It is unknown if the results from commercial IGRAs that are approved for the diagnosis of TB and LTBI correlate with clinical outcomes or follow a specific pattern after antibiotic treatment for *M. tb *infection. 

The current published data on the effects of LTBI treatment on IFN-*γ* levels are inconsistent. Studies from low-transmission settings where repeat exposure to *M. tb *is unlikely have found that T-cell IFN-*γ* levels decline after treatment. One such study in the United Kingdom followed the T-cell responses in students exposed to *M. tb* in a point-source school TB outbreak. Students meeting the UK guideline for the treatment of LTBI by indication from TST results were given both rifampin (R) and H; after the completion of therapy, the levels of IFN-*γ* response in the students substantially decreased. However, the levels of IFN-*γ* response also decreased in the students who tested negative by TST and did not undergo treatment [24]. Another study in recent immigrants to the UK showed that T cells produced higher levels of IFN-*γ* one month after the initiation of treatment for LTBI but towards the end of the LTBI treatment course, the IFN-*γ* level decreased. This study found that the T-cell response did not change in those with LTBI who were not initiated on treatment. Also, the author showed that peripheral blood mononuclear cells infected with *M. tb *and treated *in vitro* with H, but not R, led to an increase in the number of IFN-*γ* producing cells. These results suggest that H acts by actively destroying the bacilli through the disruption of the cell wall. Such a process may contribute to the increased release of cell wall-associated antigens, resulting in an increased number of antigens-specific T cells being detected during treatment [15].

Pai et al. (2006) have demonstrated that the baseline of T-cell IFN-*γ* response among Indian health care workers in a high-transmission setting was high even ten months after the completion of treatment for LTBI [10]. On the other hand, a study by Higuchi et al. (2008) of individuals who had contact with TB patients in Tokyo showed a decline in IFN-*γ* levels six months after the completion of treatment for LTBI without test reversion [11]. A study conducted in Rome, also an area of low transmission, found a significant decrease in IFN-*γ* levels in patients after 1-2 months of LTBI treatment and found that response of the majority of the patients became undetectable after six months [16].

Here we found that the IFN-*γ* levels of individuals with documented LTBI increased after H treatment. Regardless of baseline QFT-IT, 25 of 26 (96%) HHC had a higher IFN-*γ* level between six and 14 months after the completion of H treatment. Individuals who tested QFT-IT positive at baseline had a greater increase in IFN-*γ* levels after treatment than did individuals who tested QFT-IT negative at baseline (Figures 3(a) and 3(b)). 

 Increased production of IFN-*γ* by T cells may be the result of a massive release of antigens when the *M. tb *bacilli are killed by H treatment [24]. This theory is supported by our finding that HHC who tested positive by QFT-IT at baseline and presumably had a higher bacillary load experienced a greater increase in IFN-*γ* levels after H therapy than those who tested negative by QFT-IT. In upregulate IFN-*γ* production [25].combination, effector memory T cells can also 

TST was unlikely to influence the baseline IGRA, since the HHC submitted the blood sample for baseline testing before the TST was administered. However, the TST given at the baseline evaluation may have interfered with the follow-up IGRA result due to TST-mediated boosting of IGRA responses which has been reported elsewhere [26, 27]. The 26 HHC in this study submitted blood for follow-up IGRA testing between 4 and 14 months after the baseline evaluation that included TST (Figure 1). A recent systematic review suggests that TST affects IGRA responses 3 days after the TST is administered, but its influence may persist for several months [28].

This study was conducted in a high-transmission setting; Salvador has one of the highest incidence rates of TB in Brazil. Therefore, it is possible that some of the HHCs were reinfected with *M. tb* after the completion of treatment and before they submitted blood samples for the follow-up examination. Moreover, follow-up examinations occurred between four and 14 months after the completion of H treatment. This long period of time increases the possibility that elevated IFN-*γ* levels are due to reinfection. It is also possible that study participants were exposed to environmental mycobacteria which share the ESAT-6 and CFP-10 genes (e.g., *M. kansasii*), and this contributed to elevated production of IFN-*γ* by T cells. On the other hand, an important aspect that should also be considered is the genetic factors, which has a key role in human phenotypic variability. Several polymorphisms have been described in genes associated with cytokine expression. These polymorphisms could also influence interindividual variability and direct IFN-*γ* production [29, 30]. 

IGRAs are not able to accurately estimate IFN-*γ* production by T cells beyond 10 IU/mL, and two of 26 HHC had IGRA results above this level in the baseline examination, and 10 of 26 HHC had IGRA results above this level in the follow-up examination. These values are likely to overestimate the IFN-*γ* production and should not be considered to be different than 10 IU/mL.

One of this study's limitations is that the medications were self-administered [20]. We assume that the HHCs who returned to the hospital each month for six months and received H refills were motivated to take the medication. However, we cannot exclude the possibility that some of the 26 HHCs included in this study did not complete the LTBI treatment according the protocol. Another limitation of this study is that less than half of the HHCs who completed H treatment submitted blood for follow-up examination by QFT-IT. There were not substantial epidemiologic differences between those HHCs who returned for the follow-up exam and those who did not. Therefore, it is unlikely that the 26 HHCs who were satisfactorily tested by two IGRAs were a biased group. Another limitation is that the single QFT-IT test for each study participant before and after the completion of H treatment were not taken at the same time. Finally, this study did not have a control group of untreated HHC with LTBI in whom the T-cell responses were monitored at the same time. Limitations in the study design and the lack of active contact-tracing programs through which to monitor individuals at high risk for progressing to active disease precluded data from being collected.

## 5. Conclusion

The data presented in this study suggest that IGRA results increase in individuals after the completion of LTBI treatment; however, conflicting data from other studies and the limitations of our study do not allow us to exclude the potential role of IGRAs in monitoring LTBI treatment. To further investigate this, larger studies in high-transmission settings must be conducted, which collect data on the T-cell responses of people with LTBI before, during, and after LTBI treatment over a longer time period.
